# Guiding principles on the education and practice of theranostics

**DOI:** 10.1007/s00259-024-06657-2

**Published:** 2024-03-08

**Authors:** Thomas N. B. Pascual, Diana Paez, Andrei Iagaru, Gopi Gnanasegaran, Sze Ting Lee, Mike Sathekge, John M. Buatti, Francesco Giammarile, Akram Al-Ibraheem, Manuela Arevalo Pardo, Richard P. Baum, Berardino De Bari, Simona Ben-Haim, Jean-Yves Blay, Anita Brink, Enrique Estrada-Lobato, Stefano Fanti, Anja Tea Golubic, Jun Hatazawa, Ora Israel, Ana Kiess, Peter Knoll, Jolanta Kunikowska, Lizette Louw, Giuliano Mariani, Siroos Mirzaei, Pilar Orellana, John O. Prior, Jean-Luc Urbain, Shrikant Vichare, Sobhan Vinjamuri, Irene Virgolini, Andrew M. Scott

**Affiliations:** 1 Department of Science and Technology, Manila, Philippines; 2 Division of Human Health, Department of Nuclear Science and Applications, International Atomic Energy Agency, Vienna, Austria; 3 Division of Nuclear Medicine and Molecular Imaging, Stanford University Medical Center, Stanford, CA USA; 4 Department of Nuclear Medicine, Royal Free London NHS Foundation Trust, London, UK; 5 Department of Molecular Imaging and Therapy, Austin Health, Melbourne, Australia; 6 Olivia Newton-John Cancer Research Institute, Melbourne, Australia; 7 School of Cancer Medicine, La Trobe University, Melbourne, Australia; 8 School of Health and Biomedicine, Royal Melbourne Institute of Technology (RMIT) University, Melbourne, Australia; 9 Department of Surgery, University of Melbourne, Melbourne, Australia; 10 Steve Biko Academic Hospital, Pretoria, South Africa; 11 University of Pretoria, Pretoria, South Africa; 12 Department of Radiation Oncology, Holden Comprehensive Cancer Center, Carver College of Medicine, University of Iowa, Iowa City, IA USA; 13 Department of Nuclear Medicine, King Hussein Cancer Center (KHCC), Amman, Jordan; 14 School of Medicine, University of Jordan, Amman, Jordan; 15 Center for Advanced Radiomolecular Precision Oncology, Curanosticum Wiesbaden, FrankfurtWiesbaden, Germany; 16 Radiation Oncology Department, Réseau Hospitalier Neuchâtelois, La Chaux-de-Fonds, Switzerland; 17 Department of Biophysics and Nuclear Medicine, Hadassah University Hospital, Jerusalem, Israel; 18 Faculty of Medicine, Hebrew University, Jerusalem, Israel; 19 University College London, London, UK; 20 Department of Medicine, Centre Leon Berard, Lyon, France; 21 University Claude Bernard Lyon, Lyon, France; 22 Nuclear Medicine Division, IRCCS Azienda Ospedaliero-Universitaria Di Bologna, Policlinico S.Orsola, Bologna, Italy; 23 Department of Nuclear Medicine and Radiation Protection, University Hospital Centre Zagreb, Kispaticeva 12, 10000 Zagreb, Croatia; 24 Department of Nuclear Medicine and Tracer Kinetics, Osaka University Graduate School of Medicine, Osaka University, Osaka, Japan; 25 B. Rappaport School of Medicine, Israel Institute of Technology-Technion, Haifa, Israel; 26 Department of Radiation Oncology and Molecular Radiation Sciences, Johns Hopkins University School of Medicine, Baltimore, MD USA; 27 Center of Molecular Imaging and Theranostics, Johannesburg, South Africa; 28 University of the Witwatersrand, Johannesburg, South Africa; 29 Regional Center of Nuclear Medicine, Department of Translational Research and Advanced Technologies in Medicine and Surgery, University of Pisa, Pisa, Italy; 30 Department of Nuclear Medicine With PET-Centre, Clinic Ottakring, Vienna, Austria; 31 Pontificia Universidad Católica, Santiago, Chile; 32 Department of Nuclear Medicine and Molecular Imaging, Lausanne University Hospital, Lausanne, Switzerland; 33 Faculty of Biology and Medicine, University of Lausanne, Lausanne, Switzerland; 34 Roswell Park Comprehensive Cancer Center, Buffalo, NY USA; 35 Nuclear Medicine Department, Liverpool University Hospitals NHS Foundation Trust, Liverpool, UK; 36 Department of Nuclear Medicine, Medical University of Innsbruck, Innsbruck, Austria; 37 Faculty of Medicine, University of Melbourne, Melbourne, Australia; 38 Nuclear Medicine Department, Medical University of Warsaw, Warsaw, Poland

**Keywords:** Theranostics, Curriculum, Education, Training

## Abstract

**Purpose:**

The recent development and approval of new diagnostic imaging and therapy approaches in the field of theranostics have revolutionised nuclear medicine practice. To ensure the provision of these new imaging and therapy approaches in a safe and high-quality manner, training of nuclear medicine physicians and qualified specialists is paramount. This is required for trainees who are learning theranostics practice, and for ensuring minimum standards for knowledge and competency in existing practising specialists.

**Methods:**

To address the need for a training curriculum in theranostics that would be utilised at a global level, a Consultancy Meeting was held at the IAEA in May 2023, with participation by experts in radiopharmaceutical therapy and theranostics including representatives of major international organisations relevant to theranostics practice.

**Results:**

Through extensive discussions and review of existing curriculum and guidelines, a harmonised training program for theranostics was developed, which aims to ensure safe and high quality theranostics practice in all countries.

**Conclusion:**

The guiding principles for theranostics training outlined in this paper have immediate relevance for the safe and effective practice of theranostics.

## Introduction

The field of theranostics represents a synergistic and dynamic convergence of specific targeted imaging and therapy, offering a paradigm shift in the management of various diseases, particularly cancers [1–10]. As this innovative approach gains momentum, the importance of a multidisciplinary approach cannot be overstated. A successful nuclear theranostics program is built upon a comprehensive foundation that encompasses advanced equipment and regulatory compliance and the collective expertise of a diverse team [11–13]. The need for the multidisciplinary team to embrace the humanistic and technical components of modern healthcare would bring the expertise of nuclear medicine physicians, other theranostics qualified cancer specialists, and professionals well-versed in evolving regulatory standards, together to form a synergistic force to navigate the intricacies of theranostics to deliver optimal patient care.

Nuclear medicine physicians and qualified specialists serve as the cornerstone of this theranostics multidisciplinary team. Their expert knowledge of unsealed radiation sources of radiopharmaceuticals used in theranostics, oversight and interpretation of molecular imaging studies, assessment and management of patients for theranostics treatment, and radiation safety requirements lays the groundwork for accurate disease identification, staging, and appropriate treatment decisions and implementation [11–15]. Working closely with nuclear medicine technologists, nurses, radiopharmaceutical scientists/radiochemists, and medical physicists ensures that the diagnostic and therapeutic phases of theranostics are executed precisely, allowing patient selection and treatment to be delivered in a timely and professional manner. Other specialists, including medical oncologists, radiation oncologists, endocrinologists, surgeons, palliative care, and pain specialists, are equally integral to this team, and the need to foster collaborative discussions among these specialists will refine treatment strategies, each contributing a unique perspective informed by their respective domains [12–15].

Navigating the evolving landscape of regulatory requirements is a shared responsibility that extends across all disciplines [12, 13, 16–18]. Regulatory bodies continuously adapt as theranostics evolve in order to ensure patient safety and quality care. The multidisciplinary team must stay abreast of these changes, integrating new guidelines seamlessly into their practice. This dynamic interplay between medical expertise and regulatory compliance is crucial, underscoring the need for ongoing education and cross-disciplinary communication.

In the practice of theranostics, where diagnostics and therapy harmonise, multidisciplinary team education, advanced equipment, and evolving regulatory adherence coalesce. The collective expertise of nuclear medicine physicians, radiation oncologists, and other cancer specialists, informed by a keen understanding of regulatory dynamics, forms the bedrock of safe and effective theranostics practice. This results in a patient-centric approach that capitalises on the transformative potential of theranostics, leading to improved outcomes and redefining the trajectory of modern nuclear medicine [11–13].

Standardised education and training programs are pivotal in ensuring a competent workforce capable of delivering high-quality theranostics nuclear medicine services. It is well known that there is significant regional diversity in nuclear medicine practice worldwide, including incorporation of modern theranostics practice [13]. Efforts have been made by major professional organisations such as the International Atomic Energy Agency (IAEA), the European Association of Nuclear Medicine (EANM), the Society of Nuclear Medicine and Molecular Imaging (SNMMI), the Australasian Association of Nuclear Medicine Specialists/ Australian and New Zealand Society of Nuclear Medicine (AANMS/ANZSNM), the International Centres for Precision Oncology (ICPO), and other professional regional groups in nuclear medicine to standardise the basic minimum requirements for training in theranostics [12, 13, 17–19] (https://www.uems.eu/__data/assets/pdf_file/0009/166977/UEMS-2023.38-European-Training-Requirements-for-the-Specialty-of-Nuclear-Medicine.pdf). Standardised education fosters uniformity and consistency in knowledge acquisition and skill development by defining core competencies, curricula, and training requirements. Establishing minimum facility requirements is essential to maintain consistency and uniformity in theranostics nuclear medicine practice. Regulatory bodies are crucial in overseeing and ensuring adherence to standardised practices within the theranostics nuclear medicine field, and standardisation through regulatory oversight establishes a framework for accountability, quality control, and continuous improvement, safeguarding patients and practitioners.

In the era of precision medicine, theranostics holds immense potential in improving patient care and treatment outcomes. However, to maximise its benefits, it is crucial to establish standardised practices across the field. An IAEA meeting was held in May 2023 with representation of experts in theranostics practice and education, aiming to establish global guidelines in theranostics training. This consensus white paper addresses the guiding principles on standardising education and training for theranostics practitioners, minimum facility requirements, and regulatory body responsibilities to streamline the theranostics nuclear medicine profession (including radiation oncology in the USA) under the scaffolding principle of social and technical health system requirements for multidisciplinary team practice. These recommendations are intended to be the minimum required for safe and effective practice of theranostics by qualified physicians, and are not intended to replace an existing comprehensive nuclear medicine training program that already addresses all of the recommendations of this white paper.

## Education and training for theranostics: building competencies for the multidisciplinary theranostics team

Building educational competence for practitioners in theranostics is fundamental in ensuring safe and effective patient care [12, 13, 17–19] (https://www.uems.eu/__data/assets/pdf_file/0009/166977/UEMS-2023.38-European-Training-Requirements-for-the-Specialty-of-Nuclear-Medicine.pdf). The significance of robust academic preparation, encompassing nuclear medicine physicians, radiation oncologists, other clinicians, and professionals, should not be understated. This comprehensive multidisciplinary approach involves establishing basic requirements and nurturing continuous professional education, ultimately fostering a proficient and adaptable workforce capable of navigating the complexities of theranostics applications. Overall, the training requirements and subsequent professional development for theranostic practitioners should allow the following outcomes:

- Emphasise the importance of proficiency in providing appropriate patient care, medical knowledge, interpersonal skills, practice-based learning and improvement, professionalism, and ethical behaviour;
- Encourage continuous professional development and acquisition of new competencies in theranostics practices for nuclear medicine specialists or appropriately qualified and credentialed physicians;
- Produce knowledgeable and competent physicians capable of adapting to advancements in the field and expanding the scope of theranostic practice.

The theranostic training curriculum proposed in this white paper aims to support Nuclear Medicine and other qualified physicians in performing radiopharmaceutical theranostic applications based on well-defined/established guiding principles (Table 1). Currently, there is variability in the quality and training pathways available worldwide for practicing radiopharmaceutical theranostics [13]. Therefore, there is a need to establish minimum basic requirements and harmonise training in this field. Standardising the training approach ensures compliance with optimal standards of theranostic care. Trainees and certified physicians are encouraged to collaborate closely with clinicians in departments where most radiopharmaceutical therapy referrals originate. This curriculum provides guidance on the role of qualified physicians, aiming to meet quality standards for training in clinical nuclear medicine imaging and radiopharmaceutical therapies. It equips physicians with the knowledge, competencies, and skills required to deliver high-quality, safe, and appropriate theranostic care. This curriculum also suggests minimum standards for training other qualified physicians for safe and effective theranostics practice. This guideline complements the Training Curriculum for Nuclear Medicine Physicians (IAEA-TECDOC-1883) published in 2019 by the IAEA [17] and the Syllabus of UEMS Educational Training Requirements for nuclear medicine physicians in Europe (https://www.uems.eu/__data/assets/pdf_file/0009/166977/UEMS-2023.38-European-Training-Requirements-for-the-Specialty-of-Nuclear-Medicine.pdf).

**Table 1:** Guiding principles on the curriculum objectives for theranostics education for physicians

Guiding principles on theranostic curriculum objectives
1. Implement a patient-centred approach by applying theoretical and practical knowledge in radiopharmaceutical theranostics, keeping in mind the fundamental principle of medicine laid down by Hippocrates: *primum non nocere*​—*FIRST DO NO HARM*​
2. Define and specify the requirements for safe delivery of high-quality theranostic services
3. Provide recommendations for minimum training requirements in the diagnostic and therapeutic aspects of using radiopharmaceuticals
4. Define the tools that will allow the training of nuclear medicine physicians and other qualified specialists that will manage patients and lead or co-lead the theranostic services
5. Outline the training required to lead and work inside a multidisciplinary professional team, including, medical physicists, radiopharmacists/radiochemists, nuclear medicine technologists, radiographers, and nurses
6. Engage trainees and nuclear medicine physicians in understanding and developing the methodology for research projects, audits, and clinical trials, which will contribute to generate evidence for the clinical use of theranostics

## Nuclear medicine physician education: curriculum requirements for theranostics practice

Nuclear medicine physicians, and/or other qualified specialists, play a central role in theranostics, requiring a deep understanding of molecular imaging, radiopharmaceuticals, radiation safety, and targeted therapies. There is a need for strong theoretical foundation in nuclear medicine, encompassing principles of radiopharmacology, imaging instrumentation, and radiation protection [12, 13, 17, 20]. Advanced learning delves into oncology, pharmacokinetics, and quantitative imaging techniques. Clinical rotations expose practitioners to diverse patient populations and imaging scenarios, honing their diagnostic skills. However, the evolving landscape of theranostics necessitates continuous learning and curriculum re-engineering. Nuclear medicine physicians and qualified specialists must stay current with emerging radiopharmaceuticals, dosimetry techniques, and cancer treatment protocols. Participation in specialised workshops, conferences, and collaborative multidisciplinary meetings ensures they remain abreast of cutting-edge developments in all aspects of disease management to ensure that theranostics is incorporated into the patient treatment plan at the appropriate juncture of the patient journey. As theranostics expands beyond oncology into areas like neurology and cardiology, a broadened curriculum becomes imperative, highlighting the interdisciplinary nature of theranostic practice.

## Basic training requirements and continuous professional education

Establishing basic educational requirements in Nuclear Medicine training [13, 14, 17] ensures a minimum level of competency across all practitioners in the field, including provisions for some training in therapy (e.g., thyroid cancer, hyperthyroidism, bone metastases). Certifications, licenses, and standardised examinations validate this proficiency, underpinned by a comprehensive curriculum covering theoretical and practical aspects. These prerequisites serve as a crucial foundation, instilling confidence in the practitioners’ abilities to deliver safe and effective theranostics.

Theranostics is a dynamic field characterised by rapid advancements. Continuous professional education becomes indispensable to maintaining competence. Regular refreshers on radiation safety protocols, dosimetry refinements, quality systems, and new technologies and radiopharmaceuticals are vital [21–23]. Interdisciplinary workshops encourage collaborative learning, fostering a shared language among diverse practitioners. Furthermore, engaging with case studies and peer-reviewed literature informs practitioners about real-world applications and challenges.

## Entry requirements and pathways for theranostics practice

1. Qualified medical practitioners/licensed physicians
  A. Nuclear Medicine physicians who have completed appropriate training and hold a license to practice nuclear medicine
  B. Qualified physicians from other specialties (including radiation oncology, radiology, and endocrinology, although not exclusively) who have completed appropriate training and are qualified and credentialed in theranostics, according to national regulations (see section below for more details)
2. Physicians undergoing training in relevant specialty (e.g., nuclear medicine, radiation oncology, radiology, endocrinology)—with appropriate training in theranostics (as below)

A comprehensive theranostics training program is crucial to equip healthcare professionals with the knowledge, skills, and confidence needed to excel in this advanced field’s diagnostic and therapeutic applications. Emphasis is recommended to include the following subject matters and competencies in the existing curriculum (Table 2). Assessment methods can follow recommendations from IAEA-TECDOC-1883 [17] or standard requirements as prescribed by individual training programs.

**Table 2:** Recommended subject matter and competencies for theranostics training

Oncology: science and practice
1. Overview of science and practice of cancer-specific standard of care (surgery, radiotherapy, chemotherapy, immunotherapy, or prior radiopharmaceutical therapies)
2. Understand basic principles and applications of molecular medicine, genetic profiling, and histopathology relevant to radiopharmaceutical therapies
3. Prognosis of different cancer subtypes and appropriate follow-up schema for disease management and response assessment
4. Understand the importance of pre-treatment clinical assessment and patient selection, including history and physical exam
5. Understand the general principles, advantages, and limitations of conventional radiological imaging (diagnosis, therapy planning, patient selection, and treatment response assessment)
6. Interpretation criteria for imaging, biochemical, pathology, and genetic results in relation to cancer subtypes
7. Attending multidisciplinary tumour boards and clinics and review of current treatment algorithms for different cancer types (including radiopharmaceutical therapies)
8. Common indications and contraindications for specific treatments (surgery, radiotherapy, chemotherapy, immunotherapy, or radiopharmaceutical therapies)
9. Common side effects and risks of specific treatments (surgery, radiotherapy, chemotherapy, immunotherapy, or radiopharmaceutical therapies)
10. Supportive care of the cancer patient, including medical, pain management, nutritional, and social services
Treatment of non-malignant conditions (hyperthyroidism, synoviorthesis, and additional indications)
1. Overview of science and management of the relevant condition and specific standard of care options (surgical, medical/systemic, and radiation therapy)
2. Pre-treatment clinical assessment, including history and physical examination
3. Interpretation of imaging, biochemical, and pathology results
4. Review of current treatment algorithms for the relevant benign conditions (including radiopharmaceutical therapies)
5. Prognosis of condition and appropriate follow-up schema for clinical management
6. Common indications and contraindications for theranostic therapy, including previous therapies and other medications
7. Side effects, toxicities, and complications related to specific treatments, including their recognition and management
8. Attending interdisciplinary boards and/or clinics
Clinical theranostics
1. General principles of molecular imaging (diagnosis, therapy planning, post-therapy imaging, and response assessment), pros and cons
2. Understand the biodistribution, pharmacokinetics, and the principles of treatment using unsealed radiation sources for theranostic radiopharmaceuticals
3. Assessment of the patient’s suitability for radiopharmaceutical therapy (clinical assessment of the patient’s medical condition, co-morbidities, performance status, and previous therapies)
4. Define the appropriate time frame for imaging in relationship to other treatments
5. Therapy response assessment criteria (imaging and non-imaging)
6. Understand informed consent, risks, benefits, and procedures of radiopharmaceutical therapies and review commonly asked questions by patients and their families related to radiopharmaceutical therapy
7. Evaluate which molecular imaging studies are appropriate for decision-making process prior to radiopharmaceutical therapy (theranostic principles)
8. Understand the indications, contraindications and potential side effects, complications, and toxicity (short term and long term) of specific radiopharmaceutical therapies
9. Patient discharge procedure and instructions for appropriate short and long-term follow-up
10. Documentation and clinical reporting
11. Knowledge and understanding of research methodology, clinical trials, clinical audits, and clinical study certification processes
Therapy specific considerations for individualised treatments
1. Indications and contraindications: absolute and relative
2. Pretherapy assessment: clinical; biochemical; imaging; molecular genomics
3. Facility and personnel requirements
4. Quality control (QC)
5. Patient preparation
6. Radiopharmaceutical preparation and administration
7. Application procedures and techniques
8. Prescribing administered activity, number of cycles, and time interval between cycles
9. Side effects and management
10. Advice upon discharge, further treatment plan and follow-up: between-cycles; intermediate and long-term follow-up, post-therapy imaging
Imaging interpretation and analysis
1. Image acquisition and imaging processing
2. Attenuation correction
3. Reconstruction
4. Recognition of pitfalls and artefacts
5. Theoretical and practical aspects of image analysis, quantitation, and dosimetry
6. Understanding the potential use of radiomics and artificial intelligence
Radiopharmaceuticals and regulatory Issues
1. Criteria for the selection of radiopharmaceuticals used in theranostics
2. Physical half-life, biological half-life, effective equivalent, and the radiation dose calculation from radiopharmaceuticals
3. Radiopharmaceutical production chain: generators and cyclotron production of radioisotopes
4. Good Manufacturing Practice (GMP) rules and in-house production processes (labelling, dispensing, QC and Quality Assurance)
5. Local Standard Operating Procedures (SOP), equipment used in the manufacturing and quality control of radiopharmaceuticals
6. Practical handling and dispensing of radioactive material
Radiation protection
1. Understanding source of radioactivity and types of exposure
2. Understanding radiation protection and following good radiation safety practices for both the staff and patient
3. Radiation dose limits and the As Low as Reasonably Achievable (ALARA) principles
4. Practical knowledge of handling and transport of radioactive material from the radiopharmacy to the patient
5. Prevention and management of contamination
6. Management of extravasation
7. Radiation protection advice for the patients, caregivers/families
8. Practical knowledge of the management of radiation safety of staff and the patient in the event of treatment complications, emergencies, or death of a patient
9. Storage and waste management
Dosimetry and radiobiology of diagnostic and therapeutic radiopharmaceuticals
1. Understanding the science and practice of internal dosimetry and radiobiology
2. Molecular and cellular effects of radiation
3. Deterministic/stochastic effects of radiation
4. Individual dose planning for specific radiopharmaceutical therapies
5. The breast-feeding patient and paediatric patients
Quality management systems
1. Regulatory and compliance requirements
2. Preparation of SOPs for therapies performed
3. Understand and develop specific radiopharmaceutical therapy care pathways
4. Clinical outcome data
Regulatory and compliance competencies
1. Understand local legal and regulatory requirements
2. Understand and operate according to departmental and hospital operation policies
3. Demonstrate interpersonal and communication skills (providing information and collaboration with patients, their families, and health professionals)
4. Maintain comprehensive, timely, and legible medical records
5. Efficient use of information technology (including artificial intelligence) to optimise learning
6. Understand health economics of theranostics

A comprehensive theranostics training program should combine didactic learning and practical experience to clinical cases. It should foster critical thinking, problem-solving, and valuable communication skills to ensure practitioners are well-prepared to navigate the intricacies of theranostics practice. In view of the rapid changes in theranostics practice and approvals, regular updates to a theranostics curriculum should be performed. Continuous professional development opportunities should be offered to stay current with advancements in the field and evolving regulatory standards. This multifaceted training approach will ultimately contribute to a confident, skilled, and proficient workforce capable of delivering high-quality theranostics care.

## Minimum procedures required to certify competence in theranostics practice

Having a minimum requirement of number of patients procedures in theranostics is essential to ensure competency and safe clinical practice of theranostics. The key criteria for minimum training and numbers of theranostic procedures for credentialing include:

1. Clinical Proficiency: A minimum requirement ensures that the individual has gained adequate hands-on experience to develop clinical proficiency in performing theranostic procedures. This expertise is crucial for accurate diagnosis and effective treatment.
2. Patient Safety: Handling a sufficient number of cases helps professionals become adept at managing potential complications and ensuring patient safety. It minimises the risk of errors and improves overall patient care.
3. Skill Development: The more patients a theranostics practitioner interacts with, the more they refine their diagnostic and therapeutic skills. This ensures that they can adapt to various clinical scenarios and provide personalised treatments.
4. Decision-Making Abilities: Experience with a diverse patient population helps in honing the ability to make informed decisions about treatment options, dosage, and follow-up care, which is critical in theranostics.
5. Quality Management: Minimum requirements help maintain a certain standard of care and quality management within the field of theranostics. It ensures that experts have demonstrated competence through a substantial number of cases.
6. Professional Accountability: It holds theranostics experts accountable for their practice and encourages continuous learning and improvement. Meeting minimum requirements demonstrates a commitment to ongoing professional development.
7. Peer Recognition: Achieving the minimum patient requirements can enhance an expert’s reputation among peers and colleagues, highlighting their dedication and expertise in theranostics.

Establishing minimum patient requirements for credentialling of theranostics experts ensures that they have the necessary competence, skill, and experience to provide high-quality patient care (Table 3). For practicing nuclear medicine physicians or qualified specialists who have performed the required number of cases during their residency/training program, and want to perform therapies or start a theranostic service, there should be a requirement of current competency in theranostics practice.

**Table 3:** Minimum required numbers of theranostics procedures for approved competence in theranostics practice*

Therapy type	Practicing Nuclear Medicine Physician or other Qualified Specialist Minimum number	Residents in-training (for the entire training period)** Recommended number
^131^I-iodide for benign thyroid disease	10	10
^131^I-iodide for thyroid cancer	20	30
Peptide Receptor Radionuclide Therapy (PRRT) in neuroendocrine tumours	20	20
Prostate Specific Membrane Antigen (PSMA) Radioligand Therapy (PRLT) for prostate cancer	20	20
Bone pain palliation (e.g., Radium-223; Strontium-89, Samarium-153, Rhenium-186; Tin-117 m)	5	5
[I^131^] mIBG for neural crest–derived tumours (for paediatrics see below)	5	5
Selective Internal Radiation Therapy (SIRT): (^90^Y resin microspheres; ^90^Y glass microspheres; ^166^Ho microspheres)	5	5
Radiosynoviorthesis Small joints: Erbium-169 (^169^Er-citrate and ^169^Er-colloids); Holmium-166 (^166^Ho-ferric hydroxide macroaggregate, or ^166^Ho-FHMA) Medium-sized joints: Rhenium-186 (^186^Re-colloids) Knee joint: Yttrium-90 (^90^Y-citrate, ^90^Y-silicate and ^90^Y-colloids) Samarium-153 (^153^Sm-particulate hydroxyapatite or ^153^Sm-PHYP)	5	5
Rhenium skin cancer therapy/epidermal radionuclide application (Rhenium-188)	5	5
Investigational/emerging theranostic procedures: radiolabelled monoclonal antibodies; alpha and Auger emitting radiopharmaceuticals; FAP peptide targeted radiopharmaceuticals; loco-regional therapies, etc	5	5
Radiopharmaceutical Therapy in Children	To be performed at an experienced centre	To be performed at an experienced centre

* Institutional involvement with individual therapies may vary, and experience may be obtained through supplemental exposure at other institutions routinely providing the treatments

**Total numbers should be minimum 100 procedures per IAEA TECDOC 1883 [17], but to practice specific areas in theranostics, the recommended minimum numbers for each procedure should be achieved

## Involvement of different professional groups in theranostics

It is recognised that there is some variation of theranostics practice worldwide, and there are potential roles of other physicians as theranostics practitioners [12, 13, 20]. The treating physician should be board-certified in nuclear medicine or a qualified and credentialed physician in theranostics, according to national regulations. The administering treating physician holds the responsibility for the safe administration of therapeutic radiopharmaceuticals and should directly supervise the process. Clinical consultation plays a vital role in evaluating a patient’s suitability for radiopharmaceutical therapy, involving a comprehensive assessment of the patient’s medical condition and appropriate molecular imaging studies. Imaging assessment guides the decision-making process regarding the suitability of radiopharmaceutical therapy for each patient, considering the timing to minimise disease progression/transformation that may affect treatment efficacy.

Beyond nuclear medicine physicians, a multidisciplinary approach brings together various clinicians and professionals. Medical oncologists, radiation oncologists, surgeons, radiologists, medical physicists, and nuclear medicine technologists all contribute their expertise. A foundational understanding of nuclear medicine principles, albeit at varying depths, is essential for these clinicians to collaborate effectively. This is evident worldwide with even more variations in theranostics practice in individual countries. For instance, medical oncologists require insights into radiopharmaceutical mechanisms, potential synergies with systemic therapies, and therapeutic dose implications. Radiation oncologists benefit from grasping the intricacies of theranostic internal dosimetry, appreciating how targeted unsealed radiation sources complement external beam therapy. Surgeons should understand pre-therapeutic imaging to inform surgical planning, ensuring optimal patient outcomes.

Multidisciplinary meetings (MDM), also known as “tumour boards” in oncology settings, are essential not only at the beginning of treatment but also at various stages, including premature cessation and complications. MDMs facilitate comprehensive discussions on imaging and clinical aspects of patient care. If treatment is recommended, the patient must be provided with practical information, logistics, potential side effects, complications, and radiation safety information relevant to their management. The treating physician or another member of the treating team delivers radiation protection instructions to the patient and family members based on national regulations and approved SOPs.

Follow-up assessments with the treating physician are conducted according to local institutional practices, including toxicity evaluations, imaging, and pathology results, to determine the patient’s suitability for further cycles of radiopharmaceutical therapy, considering any necessary dose modifications. Shared care may involve visits to various clinics.

With the expansion of theranostics to other fields of medicine beyond oncology in the future, similar multidisciplinary groups and meetings should be established and involve relevant specialty clinicians.

## Theranostics Training Centres

Establishing a Theranostics Training Centre necessitates the presence of qualified personnel, appropriate equipment, suitable teaching facilities, and a minimum annual performance of diverse diagnostic and therapeutic (theranostics) procedures (Table 4) as well as a strong quality assurance and improvement program, and scientific research, participation in clinical trials and scientific publications. Such resources enhance patient care and ensure a safe environment while facilitating access to crucial radiopharmaceuticals. Adequate equipment providing high-resolution images that guide treatment decisions and therapeutic planning including Positron Emission Tomography (PET/CT, PET/MRI) which are essential for accurate diagnosis and staging, and Single-Photon Emission Computed Tomography (SPECT/CT) which is essential for dosimetry must be available at a training centre. In theranostics, where personalised treatment is paramount, precise diagnostic information is a cornerstone. Properly maintained equipment ensures patients receive the most accurate diagnoses, enabling tailored therapeutic interventions.

**Table 4:** Personnel requirements for establishing a Theranostics Training Center

Personnel (applicable to all categories):
• Treating theranostics physician*
• Nuclear medicine technologist*
• Medical physicist with expertise in internal dosimetry*
• Radiation safety officer*
• Radiopharmaceutical scientist
• Nurse/nurse practitioner*
• Administrative and support staff*

*Mandatory

Equally crucial is having infrastructure that supports the administration of radiopharmaceutical therapies. Specialised facilities, such as dedicated infusion rooms and inpatient treatment rooms with appropriate radiation shielding, are essential for safely delivering radiopharmaceutical therapies. These facilities minimise radiation exposure to patients and healthcare workers while ensuring the therapies are effectively administered. Adequate infrastructure and protocols prevent accidental radiation exposure and contribute to safe and effective treatment. Some centres have the necessary infrastructure for radiopharmaceutical production and quality control (https://www.uems.eu/__data/assets/pdf_file/0009/166977/UEMS-2023.38-European-Training-Requirements-for-the-Specialty-of-Nuclear-Medicine.pdf.) [24–26]. This is particularly vital in theranostics, where the availability of specific radiopharmaceuticals is critical, and recognising that supply of radiopharmaceuticals from licensed radiopharmacies to a theranostics site is also an acceptable approach for theranostics practice. Access to radiopharmaceuticals that enable both diagnostic imaging and targeted therapy is a defining feature of theranostics. The minimum requirements for a Theranostics Training Centre is outlined in Table 5, recognising that different levels of clinical activity impact on suitability for training in clinical practice of Theranostics.

**Table 5:** Minimum requirements for a Theranostics Training Centre

Category 1. Basic clinical Theranostics Training Centre
• Administer at least one type of radiopharmaceutical therapy • Conduct a minimum of 100 consultations and/or administrations of radiopharmaceutical therapies per year
Category 2. Clinical Theranostics Training Centre
• Administer multiple types of radiopharmaceutical therapy • Conduct a minimum of 100 consultations and/or administrations of radiopharmaceuticals therapies per year • Perform at least 10 PRLT and/or 10 PRRT consultations and/or administrations per year • Regular conduct of therapy-based multidisciplinary meetings
Category 3. Advanced Theranostics Training Centre
• Administer multiple types of radiopharmaceutical therapy • Conduct a minimum of 150 consultations and/or administrations of radiopharmaceutical therapies per year • Perform at least 20 PRLT and/or 20 PRRT consultations and/or administrations per year • Regular conduct of therapy-based multidisciplinary meetings • Participate in recognised research in the field

The minimum recommended institutional requirements in Table 5 must also be subject to local regulatory compliance requirements.

A well-equipped and staffed theranostics facility enhances the patient experience. Patients can receive diagnostic imaging and therapeutic interventions in one location, minimising the need for multiple appointments and travel. Adequate infrastructure ensures that patients receive care within a comfortable and patient-centred environment, contributing to overall satisfaction and adherence to treatment plans. Private facilities and hospitals must adhere to stringent regulatory guidelines governing the use of radioactive materials and radiation-emitting equipment. Having adequate infrastructure ensures compliance with these regulations, safeguarding patient and staff safety. Properly designed shielding, radiation monitoring systems, and radiation safety training for staff members are all critical components of maintaining a safe environment for theranostics practice.

## Regulatory considerations for the implementation of theranostics training programs

Theranostics practice involves using unsealed radioactive materials and radiation-detecting equipment, making it subject to a complex framework of legislation, regulatory requirements, and approvals. This framework is designed to ensure patient safety, staff safety, and proper management of radioactive waste. Local regulations and legislative compliance will always take precedence over the suggested recommendations. Regulatory bodies at the national and international levels play a crucial role in overseeing and enforcing these requirements. Organisations like the IAEA, EANM, SNMMI, ANZSNM, UEMS contribute to developing international guidelines and standards for nuclear medicine practices, including theranostics. The following guiding principles are to assist the regulatory framework and ensure that there is safety, availability, and access for patients to theranostic procedures and to facilitate training programs (Table 6).

**Table 6:** Guiding principles for theranostics regulatory frameworks

Equitable access and availability of radiopharmaceutical therapy
• Equity of access should be the guiding principle for patient access to theranostics • There is a wealth of knowledge covering the current use and anticipated demand of growth in workforce, equipment access and availability of theranostic services globally [5, 10, 13, 27] • Access to radiopharmaceuticals is affected by supply chain and local capacity for production, treatment, and regulatory approvals for use [10, 13, 22, 27–31] • Provision of appropriate imaging and therapy is also dependent on a trained workforce [12, 13, 17, 18] • Access to good diagnostic testing/imaging is crucial in patient selection and monitoring response to therapy • Regulatory approval for the appropriate and safe use of theranostics procedures is subject to local and national rules as applicable • Reimbursement, either at governmental level or health insurance approval, will also dictate local availability
Regulatory approvals for theranostics require evidence and multi-centre trials
• Theranostics should follow pathways for regulatory approval and reimbursement that has been used for existing and established nuclear medicine imaging and therapeutic procedures [5, 10, 12, 18] • Some countries will recognise approvals by other jurisdictions, whereas many countries require new submission for approval of new procedures • It is recognised that the development of theranostics may not be totally dependent on industry, and in this context approaches to regulatory approvals and reimbursement need to be adapted to achieve successful outcomes • Generation of data relating to safety and efficacy of novel diagnostic and therapeutic nuclear medicine procedures is crucial in order to obtain regulatory approval • Investigator initiated trials are particularly important for the introduction of novel diagnostic and therapeutic procedures as not all trials may be sponsored by industry [10, 28, 31] • Health Technology Assessments for nuclear medicine imaging and therapeutics may require a cross-country approach in order to obtain approval and reimbursement in many countries [27] • IAEA and World Health Organisation should have an important role in supporting the adoption of nuclear medicine imaging and therapeutic procedures as part of government policy • Local, legislative, and regulatory requirements for the introduction and use of radiopharmaceuticals need to be followed • Incorporation of new diagnostic and therapeutic procedures should constitute an integral part of clinical practice guidelines, leading to their adoption into clinical practice [5, 10, 27] • Mechanisms for compassionate use and early access are important for the introduction of new procedures that have been approved in other countries
Regulatory frameworks for theranostics radiation protection of patients, staff, and public, and waste management
• Radiation safety and protection of all staff is essential, usually based on existing guidelines • The increased scope and scale of projected theranostics procedures require special attention for all aspects of radiation protection, safety, environmental impact, and waste disposal • Theranostics facilities will require careful design to address requirements to deal with higher levels of radioactivity and patient numbers, which have an impact on workflow, staff and public radiation exposure, as well as waste management • A close monitoring of staff exposure is necessary with the introduction of novel theranostic procedures [32] • Patients should be appropriately informed about the procedure and radiation protection precautions to be taken by themselves, family, caregivers, and members of the public, as well as the exact duration of such precautions [32] • Efficient and effective waste management is a critical aspect of maintaining safety for the use of radiopharmaceuticals for therapy, including both alpha and beta radionuclides [33]. Special consideration should be placed on subsequent patient related events including surgical and medical intervention, intensive care admission, and death, post theranostics procedures

The regulatory framework for theranostics radiopharmaceutical approval is critical to ensuring patient safety, equitable access, and practical implementation in clinical practice, especially in developing countries. This framework involves various processes, including radiopharmaceutical development, clinical trials, health technology assessment (HTA), and regulatory approvals. Each country has its own set of laws and regulations governing nuclear medicine practices, which encompasses theranostics. This includes guidelines for inpatient treatment based on radiation dose, disposal of waste, and staff and public radiation exposure limits. These laws also define the legal framework for possessing, using, and disposing of radioactive materials, radiation-emitting devices, and medical equipment. Regulatory bodies, such as nuclear regulatory commissions or health ministries, enforce these laws. Facilities that intend to provide theranostics services must obtain licenses or authorisations from these regulatory bodies. These licenses outline the scope of practice, equipment specifications, and safety protocols. Regulatory requirements include radiation safety measures to protect patients from unnecessary radiation exposure.

The IAEA establishes safety standards and guidelines that member states are encouraged to adopt. These standards cover radiation protection, patient dose optimisation, and quality assurance [32–34]. This involves setting maximum permissible radiation doses, establishing protocols for patient positioning, and monitoring radiation levels during procedures. Developing countries may require capacity building and technology transfer to implement theranostics effectively. Collaborative efforts between regulatory authorities, international organisations, and experts from developed countries can facilitate knowledge exchange, training, and local infrastructure development.

## Summary

In the era of precision medicine, theranostics is the integration of diagnostic and therapeutic approaches in nuclear medicine using unsealed radiation sources, and holds immense potential in improving patient care and treatment outcomes. This consensus white paper highlights guiding principles required for training and credentialling of theranostics physicians, and establishing Theranostics Training Centres. This will facilitate the access and availability of theranostics globally, and improve patient outcomes through a multidisciplinary theranostics team approach. By utilising the collective expertise of nuclear medicine physicians and other cancer specialists, as well as others in the multidisciplinary theranostics team, this will form the l framework required for a patient-centric approach to the practice of theranostics in the future.

## References

[1] Baum RP (Handbook Editor) Therapeutic nuclear medicine. Springer-Verlag Berlin Heidelberg 2014. eBook ISBN: 978–3–540–36719–2.

[2] Langbein T, Weber WA, Eiber M. Future of theranostics: an outlook on precision oncology in nuclear medicine. J Nucl Med. 2019;60(Suppl 2):13S-19S. 31481583 10.2967/jnumed.118.220566

[3] Filippi L, Chiaravalloti A, Schillaci O, Cianni R, Bagni O. Theranostic approaches in nuclear medicine: current status and future prospects. Expert Rev Med Devices. 2020;17(4):331–43. 32157920 10.1080/17434440.2020.1741348

[4] Gomes Marin JF, Nunes RF, Coutinho AM, Zaniboni EC, Costa LB, Barbosa FG, Queiroz MA, Cerri GG, Buchpiguel CA. Theranostics in nuclear medicine: emerging and re-emerging integrated imaging and therapies in the era of precision oncology. Radiographics. 2020;40(6):1715–40. 33001789 10.1148/rg.2020200021

[5] Herrmann K, Schwaiger M, Lewis JS, et al. Radiotheranostics: a roadmap for future development. Lancet Oncol. 2020;21(3):e146–56. 32135118 10.1016/S1470-2045(19)30821-6 PMC7367151

[6] Jokar N, Assadi M, Yordanova A, Ahmadzadehfar H. Bench-to-bedside theranostics in nuclear medicine. Curr Pharm Des. 2020;26(31):3804–11. 32067609 10.2174/1381612826666200218104313

[7] Buatti JM, Kiess AP. The rapid evolution of theranostics in radiation oncology. Semin Radiat Oncol. 2021;31:1–2. 33246630 10.1016/j.semradonc.2020.07.001

[8] Vahidfar N, Eppard E, Farzanehfar S, Yordanova A, Fallahpoor M, Ahmadzadehfar H. An impressive approach in nuclear medicine: theranostics. PET Clin. 2021;16(3):327–40. 34053577 10.1016/j.cpet.2021.03.011

[9] Al-Ibraheem A, Abdlkadir A, Albalooshi B, Muhsen H, Haidar M, et al. Theranostics in the Arab world; achievements & challenges. Jordan Med J. 2022;56:188–205.

[10] Bodei L, Herrmann K, Schöder H, Scott AM, Lewis JS. Radiotheranostics in oncology: current challenges and emerging opportunities. Nat Rev Clin Oncol. 2022;19(8):534–50. 35725926 10.1038/s41571-022-00652-y PMC10585450

[11] Weber WA, Czernin J, Anderson CJ, Badawi RD, Barthel H, Bengel F, Bodei L, Buvat I, DiCarli M, Graham MM, Grimm J, Herrmann K, Kostakoglu L, Lewis JS, Mankoff DA, Peterson TE, Schelbert H, Schöder H, Siegel BA, Strauss HW. The future of nuclear medicine, molecular imaging, and theranostics. J Nucl Med. 2020;61(Suppl 2):263S-272S. 33293447 10.2967/jnumed.120.254532

[12] Lee ST, Emmett L, Pattison DA, Hofman MS, Bailey DL, Latter MJ, Francis RJ, Scott AM. The importance of training, accreditation and guidelines for the practice of theranostics: the Australian perspective. J Nucl Med. 2022;63(6):819–22. 35393349 10.2967/jnumed.122.263996

[13] Urbain JL, Scott AM, Lee ST, Buscombe J, Weston C, Hatazawa J, Kinuya S, Singh B, Haidar M, Ross A, Lamoureux F. Theranostic radiopharmaceuticals: a universal challenging educational paradigm in nuclear medicine. J Nucl Med. 2023;64(6):986–91. 37142302 10.2967/jnumed.123.265603

[14] Bodei L, Chiti A, Modlin IM, Scott AM, Schoder H. The path to the future: education of nuclear medicine therapeutic specialists as responsible physicians. J Nucl Med. 2019;60:1663–4. 31732675 10.2967/jnumed.119.232454

[15] Buatti JM, Pryma DA, Kiess AP, Mailman J, Ennis RD, Menda Y, White GA, Pandit-Taskar N. A Framework for patient-centered pathways of care for radiopharmaceutical therapy: an ASTRO consensus document. Int J Radiat Oncol Biol Phys. 2021;109(4):913–22. 33249143 10.1016/j.ijrobp.2020.11.048

[16] International Atomic Energy Agency, “Radiation protection and safety of radiation sources: international basic safety standards general safety requirements part 3”, IAEA Safety Standards Series No. GSR Part 3, IAEA, Vienna, 2014.

[17] International Atomic Energy Agency. “Training curriculum for nuclear medicine physicians”, IAEA-TECDOC-1883. Vienna: IAEA; 2019.

[18] Herrmann K, Giovanella L, Santos A, Gear J, Kiratli PO, Kurth J, Denis-Bacelar AM, Hustinx R, Patt M, Wahl RL, Paez D. Joint EANM, SNMMI and IAEA enabling guide: how to set up a theranostics centre. Eur J Nucl Med Mol Imaging. 2022;49(7):2300–9. 35403861 10.1007/s00259-022-05785-x PMC9165261

[19] Al-Ibraheem A. Theranostics in developing countries: addressing challenges and potentials from training to practice. World J Nucl Med. 2023;22(3):171–3. 37854085 10.1055/s-0043-1774733 PMC10581750

[20] Kiess AP, Hobbs RF, Bednarz B, Knox SJ, Meredith R, Escorcia FE. ASTRO’s framework for radiopharmaceutical therapy curriculum development for trainees. Int J Radiat Oncol Biol Phys. 2022;113(4):719–26. 35367328 10.1016/j.ijrobp.2022.03.018

[21] International Atomic Energy Agency. “Good practice for introducing radiopharmaceuticals for clinical use”, IAEA-TECDOC-1782. Vienna: IAEA; 2016.

[22] International Atomic Energy Agency (IAEA). QUANUM 3.0: an updated tool for nuclear medicine audits. IAEA, Vienna. 2021.

[23] International Atomic Energy Agency (IAEA). Nuclear Medicine Resources Manual 2020 Edition. IAEA, Vienna, 2020.

[24] Annex 2: International Atomic Energy Agency and World Health Organization guideline on good manufacturing practices for radiopharmaceutical products. WHO Tech Rep Ser. 2020;1025:93–108.

[25] Gillings N, Hjelstuen O, Ballinger J, Behe M, Decristoforo C, Elsinga P, et al. Guideline on current good radiopharmacy practice (cGRPP) for the small-scale preparation of radiopharmaceuticals. EJNMMI Radiopharm Chem. 2021;6(1):8. 33580358 10.1186/s41181-021-00123-2 PMC7881071

[26] American College of Radiology, American College of Nuclear Medicine, American Society for Radiation Oncology, Society of Nuclear Medicine and Molecular Imaging. ACR-ACNM-ASTRO-SNMMI Practice Parameter for the Performance of Therapy with Unsealed Radiopharmaceutical Sources. ACR, ACNM, ASTRO, SNMMI; 15; Available at: https://www.acr.org/-/media/ACR/Files/Practice-Parameters/unsealedsources.pdf. Accessed September, 2023.

[27] Hricak H, Abdel-Wahab M, Atun R, Lette MM, Paez D, Brink JA, et al. Medical imaging and nuclear medicine: a Lancet Oncology Commission. Lancet Oncol. 2021;22(4):e136–72. 33676609 10.1016/S1470-2045(20)30751-8 PMC8444235

[28] Czernin J, Fanti S, Meyer PT, Allen-Auerbach M, Hacker M, Sathekge M, Hicks R, Scott AM, Hatazawa J, Yun M, Schoder H, Bartenstein P, Herrmann K. Nuclear medicine operations in the times of COVID-19: strategies, precautions, and experiences. J Nucl Med. 2020;61(5):626–9. 32238430 10.2967/jnumed.120.245738

[29] Paez D, Gnanasegaran G, Fanti S, Bomanji J, Hacker M, Sathekge M, Bom H, Cerci J, Chiti A, Hermann K, Scott AM, El-Haj N, Estrada E, Pellet O, Orellana P, Giammarile F, Abdel-Wahab M. COVID-19 pandemic: guidance for nuclear medicine departments. Eur J Nucl Med Mol Imaging. 2020;47(7):1615–9. 32296886 10.1007/s00259-020-04825-8 PMC7159284

[30] Cutler CS, Bailey E, Kumar V, Schwarz SW, Bom H-S, Hatazawa J, Paez D, Orellana P, Louw L, Mut F, Kato H, Chiti A, Frangos S, Fahey F, Dillehay G, Oh SJ, Lee DS, Lee ST, Nunez-Miller R, Bandhopadhyaya G, Pradhan PK, Scott AM. Global issues of radiopharmaceutical access and availability: a nuclear medicine global initiative project. J Nucl Med. 2021;62(3):422–30. 32646881 10.2967/jnumed.120.247197 PMC8049350

[31] Francis RJ, Bailey DL, Hofman MS, Scott AM. The Australasian Radiopharmaceutical Trials Network (ARTnet)—clinical trials, evidence and opportunity. J Nucl Med. 2021;62(6):755–6. 33384324 10.2967/jnumed.120.258152 PMC8729868

[32] International Atomic Energy Agency. Radiation protection and safety in medical uses of ionizing radiation. IAEA Safety Standards Series No. SSG-46, IAEA, Vienna. 2018.

[33] International Atomic Energy Agency. Management of radioactive waste from the use of radionuclides in medicine. IAEA-TECDOC-1805, Vienna. 2017.

[34] International Atomic Energy Agency. Management of patient dose in nuclear medicine. IAEA Radiation Protection Series No. 9, Vienna. 2008.

