# Correction to: Guiding principles on the education and practice of theranostics

**DOI:** 10.1007/s00259-025-07369-x

**Published:** 2025-05-30

**Authors:** Thomas N. B. Pascual, Diana Paez, Andrei Iagaru, Gopi Gnanasegaran, Sze Ting Lee, Mike Sathekge, John M. Buatti, Francesco Giammarile, Akram Al‑Ibraheem, Manuela Arevalo Pardo, Richard P. Baum, Berardino De Bari, Simona Ben‑Haim, Jean‑Yves Blay, Anita Brink, Enrique Estrada‑Lobato, Stefano Fanti, Anja Tea Golubic, Jun Hatazawa, Ora Israel, Ana Kiess, Peter Knoll, Jolanta Kunikowska, Lizette Louw, Giuliano Mariani, Siroos Mirzaei, Pilar Orellana, John O. Prior, Jean‑Luc Urbain, Shrikant Vichare, Sobhan Vinjamuri, Irene Virgolini, Andrew M. Scott

**Affiliations:** 1 Department of Science and Technology, Manila, Philippines; 2 Division of Human Health, Department of Nuclear Science and Applications, International Atomic Energy Agency, Vienna, Austria; 3 Division of Nuclear Medicine and Molecular Imaging, Stanford University Medical Center, Stanford, CA USA; 4 Department of Nuclear Medicine, Royal Free London NHS Foundation Trust, London, UK; 5 Department of Molecular Imaging and Therapy, Austin Health, Melbourne, Australia; 6 Olivia Newton-John Cancer Research Institute, Melbourne, Australia; 7 School of Cancer Medicine, La Trobe University, Melbourne, Australia; 8 School of Health and Biomedicine, Royal Melbourne Institute of Technology (RMIT) University, Melbourne, Australia; 9 Department of Surgery, University of Melbourne, Melbourne, Australia; 10 Steve Biko Academic Hospital, Pretoria, South Africa; 11 University of Pretoria, Pretoria, South Africa; 12 Department of Radiation Oncology, Holden Comprehensive Cancer Center, Carver College of Medicine, University of Iowa, Iowa City, IA USA; 13 Department of Nuclear Medicine, King Hussein Cancer Center (KHCC), Amman, Jordan; 14 School of Medicine, University of Jordan, Amman, Jordan; 15 Center for Advanced Radiomolecular Precision Oncology, Curanosticum Wiesbaden, FrankfurtWiesbaden, Germany; 16 Radiation Oncology Department, Réseau Hospitalier Neuchâtelois, La Chaux‑de‑Fonds, Switzerland; 17 Department of Biophysics and Nuclear Medicine, Hadassah University Hospital, Jerusalem, Israel; 18 Faculty of Medicine, Hebrew University, Jerusalem, Israel; 19 University College London, London, UK; 20 Department of Medicine, Centre Leon Berard, Lyon, France; 21 University Claude Bernard Lyon, Lyon, France; 22 Nuclear Medicine Division, IRCCS Azienda Ospedaliero-Universitaria Di Bologna, Policlinico S.Orsola, Bologna, Italy; 23 Department of Nuclear Medicine and Radiation Protection, University Hospital Centre Zagreb, Kispaticeva 12, 10000 Zagreb, Croatia; 24 Department of Nuclear Medicine and Tracer Kinetics, Osaka University Graduate School of Medicine, Osaka University, Osaka, Japan; 25 25 B. Rappaport School of Medicine, Israel Institute of Technology-Technion, Haifa, Israel; 26 Department of Radiation Oncology and Molecular Radiation Sciences, Johns Hopkins University School of Medicine, Baltimore, MD USA; 27 Center of Molecular Imaging and Theranostics, Johannesburg, South Africa; 28 University of the Witwatersrand, Johannesburg, South Africa; 29 Regional Center of Nuclear Medicine, Department of Translational Research and Advanced Technologies in Medicine and Surgery, University of Pisa, Pisa, Italy; 30 Department of Nuclear Medicine With PET‑Centre, Clinic Ottakring, Vienna, Austria; 31 Pontificia Universidad Católica, Santiago, Chile; 32 Department of Nuclear Medicine and Molecular Imaging, Lausanne University Hospital, Lausanne, Switzerland; 33 Faculty of Biology and Medicine, University of Lausanne, Lausanne, Switzerland; 34 Roswell Park Comprehensive Cancer Center, Buffalo, NY USA; 35 Nuclear Medicine Department, Liverpool University Hospitals NHS Foundation Trust, Liverpool, UK; 36 Department of Nuclear Medicine, Medical University of Innsbruck, Innsbruck, Austria; 37 Faculty of Medicine, University of Melbourne, Melbourne, Australia; 38 Nuclear Medicine Department, Medical University of Warsaw, Warsaw, Poland

**Correction to: European Journal of Nuclear Medicine and Molecular Imaging (2024) 51:2320–2331**

10.1007/s00259-024-06657-2

The authors regret that they missed to include Dr. Jolanta Kunikowska and her affiliation “Nuclear Medicine Department, Medical University of Warsaw, Poland” in the author list of the original published article. She is now included in the author list of this erratum article.

The original article has been corrected.

